# Surface freezing of water

**DOI:** 10.1186/s40064-016-2196-3

**Published:** 2016-05-16

**Authors:** J. L. Pérez-Díaz, M. A. Álvarez-Valenzuela, F. Rodríguez-Celis

**Affiliations:** Departamento de Teoría de la Señal, Universidad de Alcalá, EPS, N-II km 33,600, 28801 Alcalá de Henares, Spain; Division of Space Technology, Department of Computer Science, Electrical and Space Engineering, Luleå University of Technology, Kiruna, Sweden; Saint George Tech Ltd., 71-75, Shelton Street, Coven Garden, London, WC2H 9JQ UK; MAG SOAR S.L., Av. de Europa, 82, 28341 Valdemoro, Spain

## Abstract

Freezing, melting, evaporation and condensation of water are essential ingredients for climate and eventually life on Earth. In the present work, we show how surface freezing of supercooled water in an open container is conditioned and triggered—exclusively—by humidity in air. Additionally, a change of phase is demonstrated to be triggered on the water surface forming surface ice crystals prior to freezing of bulk. The symmetry of the surface crystal, as well as the freezing point, depend on humidity, presenting at least three different types of surface crystals. Humidity triggers surface freezing as soon as it overpasses a defined value for a given temperature, generating a plurality of nucleation nodes. An evidence of simultaneous nucleation of surface ice crystals is also provided.

## Background

Nakaya demonstrated that snow crystals grow in cold humid air as a large variety of mainly plane crystals depending on both temperature and humidity of air 80 years ago (Magono and Woo 1966). As they were formed by inverse sublimation from vapor nobody got surprised that humidity—i.e. water vapor concentration in air—had a role on the kind of crystal grown. Up to 35 meteorological groups were described. More recently this number has grown up to 121 categories (Kikuchi et al. 2013).

More recently 2D confined water and ice crystals have attracted the attention of researchers. They are described to appear between two solid layers (Chen et al. 2015) with a structure depending on pressure and width (Zangi and Mark 2003). Molecular dynamics simulations show multiple possible structures of water confined between parallel bonding walls: dodecagonal quasicrystal bilayers (Johnston et al. 2010), ferroelectric hexagonal monolayers, rhombic monolayers (Zhao et al. 2014) and room temperature square ice (Algara-Siller et al. 2015). Additionally, some structural similarities between supercooled water and confined ice layers have also recently been described (Ricci et al. 2009). Particularly first neighboring oxygens and H-bonds in supercooled water and confined ice are both shorter than in common water or unconfined ice.

Hydrophobicity and hydrophilicity of surfaces confining liquid water seem to modulate its density at the nano-scale (Giovambattista et al. 2007), presenting a larger diffusivity when confined in mesopores by hydrophobic surfaces (Aso et al. 2012) and additionally “ab initio” computations demonstrate a phase change in nano-confined ice from a honeycomb to square under pressure depending on the hydrophobicity/hydrophilicity of such surfaces (Corsetti et al. 2016).

In previous works we demonstrated that humidity strongly affected surface energy, i.e. surface tension, (Pérez-Díaz et al. 2012) of water as well the freezing point of supercooled water droplets (Pérez-Díaz et al. 2015; Perez-Diaz et al. 2016). Moreover, the formation of icicles protruding from droplets suggested that surface freezing preceded bulk freezing.

However, it remained unknown whether it was a phenomenon associated to the liquid water–air interface or to the size and shape of the droplets used in those experiments. Moreover, it was no clear how freezing progressed into the bulk or if a truly surface crystallization occurred. In the present work, we aim to answer these two last questions performing new experiments with deionized water in open containers. The liquid water–air interface in these containers is perfectly flat except for the border in contact with their walls.

## Experimental method

**Fig. 1:**
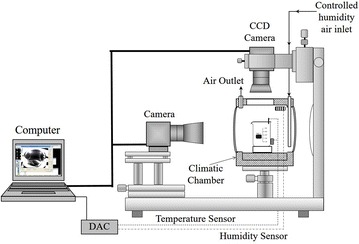
Experimental layout

A 200 ml glass container filled with colourless and odourless deionized liquid water, with a density of 1.000 ± 0.005 g/ml at 20.0 ± 0.2 °C according to the standard UNE 26-389 and an alkaline pH of 6–7.5 according to the standard UNE 26-390. Its electrical conductivity was less than 800 µS/m. The deionized liquid water was kept in a climatic chamber at atmospheric pressure (93.5 ± 0.1 kPa) specifically design to control both temperature from −13.0 ± 0.1 up to 30.0 ± 0.1 °C and humidity from 5 ± 1 to 99 ± 1 %. A schematic of the experimental layout is shown in Fig. 1. A typical operation procedure consists on the following steps: starting from room conditions, first, relative humidity is lowered down to around 13 %. Second, temperature is lowered down to a fixed valued (below 0 °C). Then, the chamber is stabilized for at least 20 min. The temperature of the supercooled water in the container becomes that of the chamber within the precision range 0.2 °C. Then humidity is increased very slowly. A top view and a side view camera are continuously monitoring the process. The image acquisition was performed in 43 full frames per second.

## Results and discussion

**Fig. 2:**
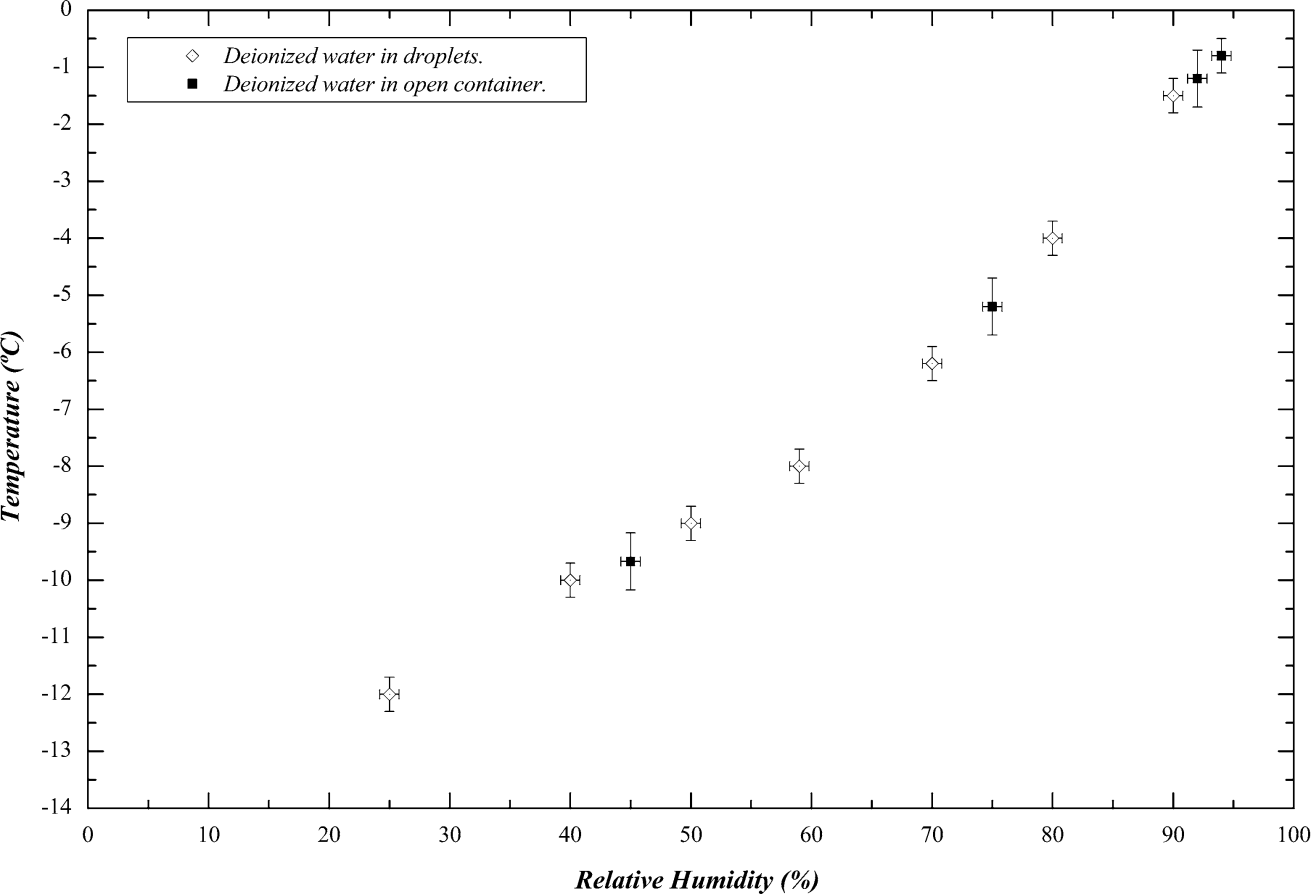
Freezing point of supercooled deionized water

In all cases freezing is triggered at the certain point as shown in Fig. 2 (black squares) wherein freezing points of water droplets previously obtained in reference (Perez-Diaz et al. 2016) have also been plotted (white rhomboids). It is evident that freezing points of water in an open container fit perfectly in the curve defined by those of droplets, demonstrating that this phenomenon does not depend on the geometry of the surface.

**Fig. 3:**
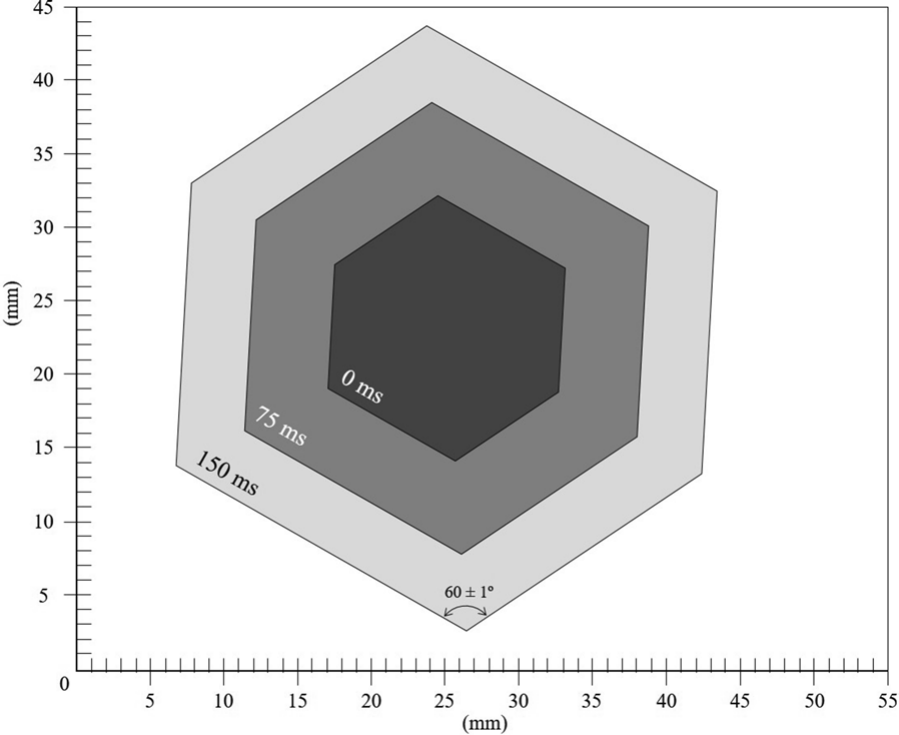
Surface crystal progression at −9.67 ± 0.1 °C and 45 ± 1 % RH for three frames every 75 ms respectively

**Fig. 4:**
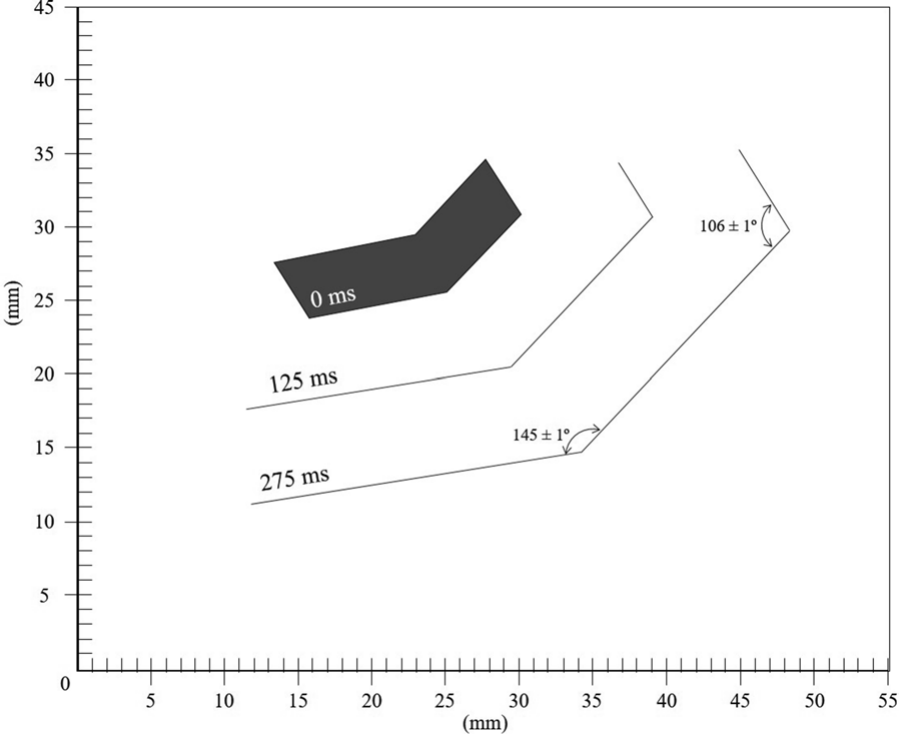
Surface crystal progression at −5.00 ± 0.10 °C and 74 ± 1 % RH for 0, 125 and 275 ms frames respectively

**Fig. 5:**
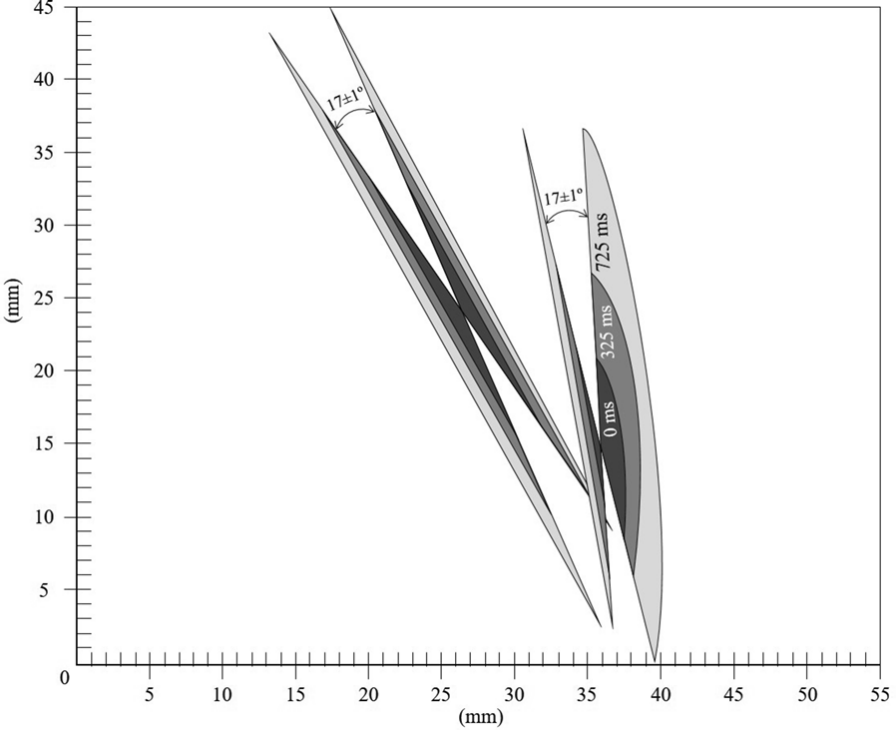
Surface crystal progression at −1.20 ± 0.1 °C and 92 ± 1 % RH for 0, 325 and 725 ms frames respectively

In all cases freezing is always nucleated at the surface. Plane single crystals are nucleated and grow on the very surface of the liquid supercooled water. Figures 3, 4 and 5 (and additional slow motion videos) show the surface crystal progression for −9.7 °C (45 % RH), −5.0 °C (74 % RH) and −1.2 °C (92 % RH). Thus, the crystals depend on humidity presenting at least three different plane phases with macroscopic symmetries apparently corresponding to wallpaper groups of p6m (hexagonal), pm (rectangular) and pmm (rectangular) respectively (Paufler 2007; Authier 2013).

Figure 5 and the corresponding video show an interesting case in which two Surface single crystals are nucleated almost simultaneously (within the 40 ms time interval between successive frames). The different apparent growth kinetics of the right branch seem to be caused by the curvature of the surface due to the surface tension and the proximity of the edge.

**Fig. 6:**
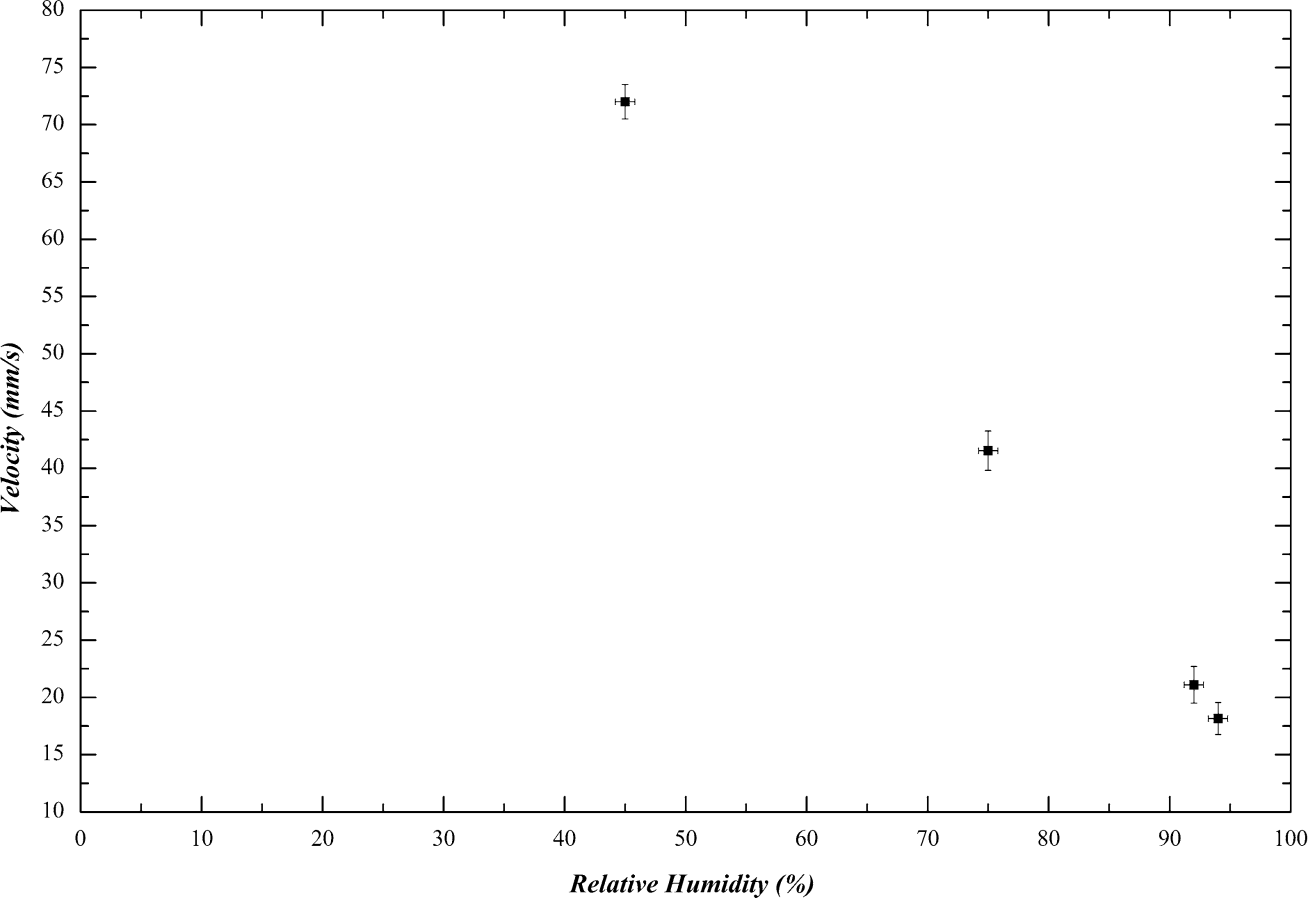
Crystal growth velocity

As it can be seen in the figures and videos above, surface crystals frontline growth velocity strongly depends on temperature (and corresponding humidity as shown in Fig. 6). The lower the temperature in the chamber, the faster the crystal grows. When the surface crystal front line reaches the wall of the container, then freezing starts progressing into the bulk.

## Conclusions

The experiments described in the present paper demonstrate that the dependence of the freezing point of deionized water on humidity in air is intrinsic to the existence of the liquid water–air interface. The freezing temperature-humidity curves for open containers and for droplets are the same. For a given temperature, surface freezing is nucleated at the liquid water–air interface by a high enough humidity, forming a surface ice crystal prior to freezing of bulk. The symmetry of the surface crystal, as well as the freezing point, depend on humidity, presenting at least three different types of surface crystals. An evidence of simultaneous nucleation of Surface ice single crystals is also provided.
